# Anti-hyperplastic effects of *Acmella oleracea* flower and leaf extracts in prostate cell lines and in spontaneously hypertensive rats

**DOI:** 10.1007/s10735-026-10736-z

**Published:** 2026-02-23

**Authors:** Edvaldo Mendes Silva, Cínthia Rio Branco da Silva, Janaína Ribeiro Costa, Aline Siqueira-Berti, Hericles Mesquita Campos, Paulo César Ghedini, Sebastião Roberto Taboga, Hernandes F. Carvalho, Mayara Tânia Pinheiro, Francisco Fábio Oliveira de Sousa, Francinaldo Sarges Braga, Roberto Messias Bezerra, Elizabeth Pereira Mendes, Manoel Francisco Biancardi, Fernanda Cristina Alcantara dos Santos

**Affiliations:** 1 Laboratory of Microscopy Applied to Reproduction, Department of Histology, Embryology and Cell Biology, Institute of Biological Sciences, Federal University of Goiás, Goiânia, Goiás 74001970 Brazil; 2 Department of Structural and Functional Biology, Institute of Biology, State University of Campinas (UNICAMP), Campinas, Brazil; 3 Laboratory of Molecular and Biochemistry Pharmacology, Department of Pharmacology, Institute of Biological Sciences, Federal University of Goiás, Goiânia, Goiás 74001970 Brazil; 4 Laboratory of Microscopy and Microanalysis, Department of Biology, University Estadual Paulista – UNESP, Rua Cristóvão Colombo, 2265, São José do Rio Preto, São Paulo 15054000 Brazil; 5 Laboratory of Biotechnology in Natural Products, Department of Biological and Health Sciences, Faculty of Pharmacy, Federal University of Amapá, Macapá, Amapá Brazil; 6 Laboratory of Quality Control, Bromatology and Microbiology, Department of Biological and Health Sciences, School of Pharmacy, Federal University of Amapá, Macapá, Amapá Brazil; 7 Atomic Absorption and Prospecting Laboratory, Federal University of Amapá, Macapá, Amapá Brazil; 8 Department of Physiological Sciences, Institute of Biological Sciences, Federal University of Goiás, Goiânia, Goiás Brazil

**Keywords:** Prostatic hyperplasia, Hypertension, RWPE-1 cells, PC-3 cells, Morphology, Endocrine modulation

## Abstract

Benign prostatic hyperplasia (BPH) is a highly prevalent age-associated disorder and a leading cause of lower urinary tract symptoms in men worldwide. Given the limitations of current therapies, there is increasing interest in phytotherapeutic compounds as sources of biologically active agents. *Acmella oleracea*, a medicinal plant rich in the alkamide spilanthol, has been traditionally associated with urogenital effects; however, the biological impact of distinct plant organs on prostate hyperplasia remains poorly defined. In this study, we investigated the effects of flower (A.Fl) and leaf (A.Le) extracts of *A. oleracea* using human prostate cell lines (RWPE-1 and PC-3) and a spontaneously hypertensive rat (SHR) model of BPH. In vitro analyses included cell viability assays and immunofluorescence for androgen receptor (AR), estrogen receptor alpha (ERα), and proliferating cell nuclear antigen (PCNA). In vivo, SHR were treated orally with A.Fl or A.Le (100 mg/kg/day for 21 days), followed by morphological, immunohistochemical, ultrastructural, and oxidative stress analyses of the ventral prostate. A.Fl displayed lower cytotoxicity than A.Le in both prostate cell lines and preferentially increased ERα immunoreactivity, whereas A.Le more strongly modulated AR without affecting cell proliferation. In SHR, both extracts attenuated prostatic hyperplasia, although A.Fl produced a more pronounced reduction in epithelial proliferation and stromal remodeling. These effects occurred independently of changes in systemic blood pressure or antioxidant activity. Collectively, these findings demonstrate that flower and leaf extracts of *A. oleracea* exert distinct biological and endocrine-modulatory effects on prostate tissue. The present data provide experimental evidence that different plant organs differentially influence epithelial-stromal dynamics and steroid receptor signaling in prostatic hyperplasia, supporting further mechanistic and translational investigations.

## Introduction

Benign prostatic hyperplasia (BPH) is a highly prevalent urological condition in aging men, affecting up to 60% of individuals between 50 and 60 years and nearly 90% of those over 80 years (Akbari et al. 2022; Gharbieh et al. 2023; Hata et al. 2023). Characterized by the proliferation of epithelial and stromal cells in the periurethral prostate, BPH leads to glandular enlargement and lower urinary tract obstruction, representing a major cause of morbidity and reduced life quality in older men (Yang et al. 2022; Wu et al. 2023).

The BPH pharmacological management commonly involves the use of α1-adrenoceptor antagonists and/or 5α-reductase inhibitors (Gul and Kaplan 2019). Surgical intervention is generally reserved for severe cases in which pharmacological therapy proves ineffective (Xia et al. 2023). Lack of response and drug resistance contribute to BPH progression, affecting more than 30% of patients (Yang et al. 2022). Therefore, the search for novel therapeutic approaches and potential pharmacological agents for the treatment of chronic urogenital disorders, such as BPH and prostate cancer, remains a major challenge for researchers in reproductive biology worldwide. In this scenario, phytotherapeutics have emerged as a promising and relevant alternative for BPH management.

*Acmella oleracea* (L.) R.K. Jansen is an Amazonian medicinal herb belonging to the Asteraceae family, also referred to as *Spilanthes acmella* var. oleracea (L.) or *Spilanthes oleracea L*. (Barbosa et al. 2016). Although native to Peru and Brazil, it is widely distributed in tropical and subtropical regions worldwide, including Bolivia, Mexico, the United States, Uganda, Kenya, Tanzania, Bangladesh, Nepal, India, Sri Lanka, Indonesia, China, and northern Australia (Dubey et al. 2013; Spinozzi et al. 2022). This species is rich in phytochemicals such as alkaloids, flavonoids, saponins, steroidal glycosides, and tannins (Lalthanpuii and Lalchhandama 2019; Spinozzi et al. 2022). The major bioactive compound in its flowers, leaves, and stems is spilanthol (Peretti et al. 2021; Rodrigues et al. 2023).

Evidence indicates that *A. oleracea* exhibits a wide range of biological activities, including analgesic, anti-inflammatory, antioxidant, antinociceptive, antifungal, diuretic, anthelmintic, and insecticidal effects (Moreno et al. 2012; Nomura et al. 2013; Paulraj et al. 2013). In addition, it has been traditionally used as an aphrodisiac in India and northern Brazil to enhance libido and sexual performance (da Rocha et al. 2018; Spinozzi et al. 2022). These attributes highlight *A. oleracea* as a species of therapeutic interest and a potential candidate for the management of prostatic diseases.

Experimental models have been fundamental for understanding prostate biology and disease mechanisms (Zhang et al. 2021; Ruiz et al. 2023). RWPE-1 cells, derived from normal human prostate epithelium, and PC-3 cells, an androgen receptor–independent prostate cancer cell line, are widely established models for in vitro investigations, allowing the evaluation of androgen-dependent and androgen-independent prostate cell responses (Moya et al. 2023; Sailer et al. 2023).

In parallel, the spontaneously hypertensive rat (SHR), widely used as a model of systemic hypertension, has also proven useful for studying BPH, as it naturally develops hyperplasia of the ventral prostate lobe concomitantly with the progression of hypertension (Saito et al. 2014; Shimizu et al. 2021; Santos et al. 2024; Seeni et al. 2025). Chronic hypertension has been directly implicated in the development of BPH through mechanisms involving reduced prostatic perfusion, tissue hypoxia, chronic inflammation, oxidative stress, and endocrine imbalance (Saito et al. 2014; Shimizu et al. 2015; Kyoda et al. 2019). Accordingly, the SHR represents a well-established and clinically relevant model for investigating BPH without the need for exogenous hormonal manipulation (Zhang et al. 2021).

Despite growing interest in phytotherapeutic approaches for BPH, the biological effects of different plant organs and their potential to differentially modulate endocrine and stromal pathways in the prostate remain poorly understood. In particular, it is unclear whether distinct phytochemical profiles derived from flowers and leaves may result in divergent effects on androgen and estrogen receptor signaling, inflammation, and epithelial-stromal interactions.

Based on this rationale, we hypothesized that flower and leaf extracts of *A. oleracea* exert distinct biological and endocrine-modulatory effects in the prostate, which may differentially influence hyperplastic progression. To test this hypothesis, the present study evaluated the effects of *A. oleracea* flower and leaf extracts in a SHR and in human prostate cell lines, integrating histological, molecular, ultrastructural, and biochemical endpoints.

## Materials and methods

### Extract production from flowers and leaves of *Acmella oleracea*

*Acmella oleracea* was cultivated and collected in the district of Fazendinha, located nine kilometers from Macapá–Amapá (Lat. 00° 02′ 30.40″ S/Long. 51° 06′ 37.5″ W). Access to and registration of the species was approved by the National System for the Management of Genetic Heritage and Associated Traditional Knowledge (SISGEN, registration A08024). Crude ethanolic extracts were obtained by hydrodistillation and individual maceration of the flowers (A.Fl) and leaves (A.Le), according to methodologies of Peretti et al. (2021) and Rodrigues et al. (2023). A.Fl and A.Le were dehydrated in an oven at 50ºC and manually fractionated after drying. The plant material was macerated in 92% ethanol for three days. The extraction procedure was repeated until complete exhaustion, which was indicated by the transparency of the solvent. The resulting macerate was then filtered by gravity and concentrated under reduced pressure using a rotary evaporator at 50 °C to remove the solvent. The extraction yield (%) was determined by comparing the weight of the crude extract to the initial weight of the dried plant material prior to extraction. The extracts were sent for analysis at the Proteomics and Mass Spectrometry Unit of the Research Infrastructure and Technology Development Network, University of Santiago de Compostela, Spain. Analyses were performed using Ultra-High-Performance Liquid Chromatography coupled with Quadrupole Time-of-Flight Mass Spectrometry (UHPLC-ESI-QTOF-MS). Briefly, both extracts were predominantly composed of the N-alkylamide spilanthol ((2E,6Z,8E)-N-isobutyl-2,6,8-decatrienamide), which accounted for approximately 97.7% of the A.Fl extract and 98.5% of the A.Le. In addition to spilanthol, the A.Fl extract contained minor constituents, including scopoletin (7-hydroxy-6-methylchromen-2-one; 1.53%) and d-limonene (1-methyl-4-(prop-1-en-2-yl)cyclohex-1-ene; 0.77%), whereas spilanthol was virtually the sole compound detected in the A.Le. Detailed analytical procedures and chromatographic profiles have been reported elsewhere (Peretti et al. 2021; Rodrigues et al. 2023).

### In vitro assays

In vitro analyses were conducted at the National Institute of Photonics Applied to Cell Biology (INFABIC/IB-UNICAMP). RWPE-1 and PC-3 cells (acquired from ATCC®; deposits CRL-11609 and CRL-1435) were cultured in Keratinocyte medium (Keratinocyte-SFM 1X, 17005042, Gibco™, ThermoFisher, US) and Ham-F12 medium (Nutrient Mixture F-12 Ham, N3520, Sigma-Aldrich, US), respectively. Both cell lines were supplemented with 10% fetal bovine serum and 1% antibiotic (penicillin–streptomycin/R30-002-CI, Corning). Cells were seeded in 96-well plates with their respective complete media. A total of 20,000 cells per well were plated (in quintuplicates) and incubated overnight at 37 °C with 5% CO₂. After removal of the culture medium, wells were supplemented with A.Fl or A.Le in incomplete medium (without serum or antibiotics) for 24 h. Both crude extracts were diluted in 100% ethanol at a concentration of 100 μg/mL. For the cell viability assay, four doses were tested: 0.2, 0.6, 1.4 and 2 μg/mL. Control cells (C) were cultured only with incomplete medium and the equivalent volume of vehicle used for extract dilution.

To evaluate cytotoxicity, the tetrazolium bromide reduction assay (MTT-3-(4,5-dimethylthiazol-2-yl)-2,5-diphenyltetrazolium bromide; Sigma/Merck, M2128) was employed. Briefly, 50 μL of MTT (5 mg/mL) was added to each well, followed by 3 h incubation. After incubation, 100 μL of dimethyl sulfoxide (DMSO) was added to solubilize the formazan crystals. Absorbance was measured at 570 nm using a microplate reader (Biotek Synergy™ HT).

RWPE-1 and PC-3 cells from groups C, A.Fl, and A.Le were subjected to immunofluorescence staining for androgen receptor (AR; rabbit polyclonal IgG, PA5-16363, Invitrogen), estrogen receptor alpha (ERα; rabbit polyclonal IgG, PA5-16,440, Invitrogen), and proliferating cell nuclear antigen (PCNA; mouse monoclonal IgG, 13-3900, Invitrogen). Cells were seeded on glass coverslips in 24-well plates and cultured overnight in complete medium. Treatments were carried out with 0.2 μg/mL of A.Fl or A.Le diluted in incomplete medium for 24 h (in quintuplicates). After exposure, cells were fixed with 4% paraformaldehyde, 4% sucrose, and 0.6% Triton X-100 for 20 min at room temperature, washed in PBS, and permeabilized with 0.5% Triton X-100 for 10 min. Non-specific binding was blocked with 3% BSA in PBS containing 0.8% Triton X-100 for 1 h. Primary antibodies (1:500 in PBS + 1% BSA) were incubated overnight at 4 °C. After PBS/Tween washes, secondary antibodies and DAPI (1:2000 in PBS+1% BSA) were incubated for 1 h, followed by PBS washes and mounting. Images were acquired using a Zeiss LSM780-NLO confocal microscope (Carl Zeiss AG, Germany). Quantification of AR and ERα was performed by optical densitometry of immunostaining intensity in 100 nuclei/group. For PCNA, the percentage of positive cells was determined from 10 photomicrographs/group. Image analyses were conducted using Fiji/ImageJ software.

### In vivo tests

#### Experimental design

A total of 32 male rats (*Rattus norvegicus*), 10 months old, were used in this experiment, comprising 24 spontaneously hypertensive rats (SHR) and 8 Wistar rats. All animals were housed in the sectorial animal facility of the Institute of Biological Sciences II in polyethylene cages with wood shavings as bedding, under controlled light and temperature conditions (23 °C), with filtered water and standard chow provided ad libitum. Animal handling and experimental procedures were approved by the Ethics Committee for Animal Use of the Federal University of Goiás (CEUA/UFG; protocol 064/20).

Wistar rats were used solely as normotensive controls for blood pressure and received only the vehicle for botanical extract dilution (200 μL saline solution) by gavage. SHR were divided into three experimental groups (*n* = 8/group): Control (C): saline solution by gavage; (A.Fl): 100 mg/kg crude ethanolic extract of *A. oleracea* flowers; (A.Le): 100 mg/kg crude ethanolic extract of *A. oleracea* leaves. The dose of 100 mg/kg/day was selected based on prior in vivo studies reporting biological activity of *A. oleracea* extracts at similar dose ranges without signs of systemic toxicity (Nomura et al. 2013; Rodrigues et al 2023). Extracts were diluted in 200 μL saline solution and administered daily for 21 days.

Blood pressure measurements by tail-cuff plethysmography were performed twice a week. Animals were warmed with an infrared light for 10 min to dilate the caudal artery. A cuff and a pulse sensor were then placed on the tail for arterial pulse detection. The system was connected to BP-100 signal amplifiers and a PowerLab/400 acquisition unit. Recordings were obtained with LabChart7 software (AD Instruments). Animals were not physically restrained during the procedure. Systolic blood pressure values were calculated as the arithmetic mean of three consecutive measurements per animal.

At the end of treatments, all animals were weighed and anesthetized with an overdose of dissociative anesthetics (xylazine 60 mg/kg and ketamine 300 mg/kg). The high doses of ketamine and xylazine were used to induce deep anesthesia prior to euthanasia, ensuring complete loss of consciousness and compliance with animal welfare guidelines. Euthanasia was performed by exsanguination, and the ventral prostate, liver, and kidneys were dissected. Organs were weighed, and the ventral prostate was processed for morphological and biochemical analyses. To assess potential extract toxicity, qualitative histopathological analysis of liver and kidney samples (*n* = 5 animals/group) was performed.

#### Histological processing, cytochemistry, and histopathology

Organs were fixed either in 4% paraformaldehyde (PFA, in Sørensen phosphate buffer, 0.1 M, pH 7.2; *n* = 3) for 24 h, or in metacarn (methanol 60%, chloroform 30%, acetic acid 10%; *n* = 5) for 4 h at 4 °C. Fixed organs were dehydrated in ethanol, cleared in xylene, and embedded in Paraplast (Histosec®, Merck®, Darmstadt, Germany). Sections (5 μm) were cut using a Leica RM2155 microtome (Leica®, Nussloch, Germany) and stained with hematoxylin and eosin (HE), periodic acid–Schiff reaction (PAS), picrosirius–hematoxylin, and toluidine blue (pH 4.0). Histological slides were analyzed and digitized using a Zeiss Axioscope A1 light microscope (Zeiss®, Germany).

#### Morphometry

All quantitative analyzes were performed by investigators blinded to the experimental groups. Stereological analyses of the prostate were performed using a multipoint test system with 130 points and 10 lines (Weibel 1963). For HE-stained sections, the relative frequency (%) of each prostatic compartment (epithelium, lumen, muscular stroma, and non-muscular stroma) was determined as described by Huttunen (1981). Thirty random microscopic fields (20 × objective) were analyzed per experimental group (*n* = 5 animals, 6 micrographs/animal). A test grid with 130 points was superimposed on each micrograph, and coincident points over each compartment were counted and expressed as relative values. For picrosirius-stained sections, the same method was applied to assess collagen content. Analyses were performed using Image Pro-Plus v6.1 for Windows (Media Cybernetics Inc., Silver Spring, MD, USA).

To quantify the area of prostatic secretion, PAS-stained sections (*n* = 5 animals/group) were digitized under constant illumination (20 × objective). PAS-positive secretion area (µm^2^) in the apical region of epithelial cells and glandular lumen was measured using Fiji (ImageJ) with the color threshold tool, according to the methodology proposed by Silva et al. (2019). The number of mast cells per microscopic field (30 random fields, 20 × objective; *n* = 5 animals/group, 6 images/animal) was obtained by counting toluidine blue–stained cells.

To evaluate the potential of botanical extracts to reduce prostatic glandular hyperplasia, the relative frequency (%) of hyperplastic alveoli was determined. Thirty histological sections from central regions of the ventral prostate were analyzed per experimental group (*n* = 8 animals). Alveoli were classified as normal (flat lining epithelium, absence of papillary projections), mildly/moderately hyperplastic (papillary projections ≤ 50% of alveolar area), or severely hyperplastic (papillary projections > 50% of alveolar area) (Santos et al. 2024). Relative frequencies of each phenotype were calculated as the proportion of alveoli in each class over the total number of alveoli per section.

#### Immunohistochemistry

Histological sections (*n* = 5/group) were processed for AR and PCNA (epithelium), and AQP1 (stroma) immunostaining. After dewaxing and rehydration, antigen retrieval was performed in citrate buffer (pH 6.0, 100 °C, 1 h). Sections were incubated with 3% H₂O₂, nonspecific protein interactions block, and primary antibodies (1:100 in 1% BSA, overnight, 4 °C): AR (rabbit polyclonal, PA5-16363, Invitrogen), PCNA (mouse monoclonal, 13-3900, Invitrogen), and AQP1 (rabbit polyclonal, PA5-78805, Invitrogen). Detection was performed using the NovoLink Polymer System (Leica Biosystems), followed by DAB staining hematoxylin counterstaining, dehydration, and mounting in Entellan (Merck).

For all immunohistochemical analyses, 30 micrographs were obtained per group (*n* = 5 animals). The total number of AR- and PCNA-positive epithelial cells was obtained as relative frequency (%) in relation to the total number of epithelial cells in each photomicrograph. For AQP1-immunolabeled sections, two complementary analyses were performed. 1—Relative frequency (%) of blood vessels per photomicrographic field: because AQP1 staining delineates blood vessels, all vessels were quantified using the multipoint test (130 points, 10 lines; Image Pro-Plus v6.1) (Weibel 1963) 2—AQP1-immunolabeled area (μm^2^): the AQP1-positive endothelial area was quantified in Fiji (ImageJ) using the color threshold tool. In this approach, only the area corresponding to AQP1-positive endothelial cells was measured per photomicrographic field.

#### Ultrastructural analysis

Ventral prostates (*n* = 3/group) were fixed in 2.5% glutaraldehyde and 0.2% picric acid in 0.1 M sodium cacodylate buffer (pH 7.2) for 24 h at 4 °C. Samples were washed twice in sodium cacodylate buffer (0.1 M, pH 7.2) and post-fixed in 1% osmium tetroxide for 2 h at 4 °C. Dehydration was performed in acetone, and samples were embedded in Epon 812 resin. Ultrathin Sections (50–70 nm) were contrasted with 2% uranyl acetate and 0.3% lead citrate. Copper grids were analyzed using a JEM-2100 transmission electron microscope (Jeol, Akishima, Japan) at the Laboratório Multiusuário de Microscopia de Alta Resolução da Universidade Federal de Goiás (LabMic/UFG).

#### Oxidative stress assays

One lobe of the ventral prostate (*n* = 8/group) was frozen in liquid nitrogen and stored at − 80 °C. Homogenization was performed in phosphate buffer (pH 7.4; ratio 1:5). After centrifugation (4000 rpm, 2 °C, 15 min), supernatants were used for the quantification of catalase (CAT) and superoxide dismutase (SOD) activities, thiobarbituric acid reactive substances (TBARS), and protein carbonyls (PC). CAT activity was determined by adding 191 µL phosphate buffer (pH 7.0), 2 µL sample (supernatant), and 7 µL H₂O₂ solution (0.3 M). The decomposition of H₂O₂, directly proportional to enzymatic activity, was monitored spectrophotometrically every 15 s for 2 min at 240 nm (Boveris and Chance 1973). SOD activity was determined in glycine–NaOH buffer (pH 10.0) with 2.5 µL homogenate in a final volume of 100 µL. Adrenaline bitartrate (1.7 µL; 60 mM, pH 2.0) was added, and absorbance was recorded spectrophotometrically at 480 nm for 10 min at 1-min intervals (Boveris et al. 1983). TBARS assay was performed to measure lipid peroxidation, following Okoh et al. (2024). Samples were incubated with thiobarbituric acid, trichloroacetic acid (pH 3.4), and SDS at 95 °C for 60 min. The reaction product was quantified spectrophotometrically at 532 nm. Results were expressed as malondialdehyde (MDA) equivalents (nmol/mg protein) based on an MDA standard curve. Protein carbonyl derivatives were measured as described by Campos et al. (2022). Samples were incubated with 2,4-dinitrophenylhydrazine (DNPH) in 2 M HCl for 1 h, with intermittent vortexing every 15 min. After sample denaturation, absorbance was measured at 370 nm.

#### Statistical analysis

Data normality was assessed using the Shapiro–Wilk test. Hypothesis testing for statistical significance was performed using Kruskal–Wallis test for nonparametric data and ANOVA for parametric data. Appropriate post hoc tests were applied following ANOVA to control for multiple comparisons. These approaches were selected to reduce the risk of type I error while maintaining sensitivity to biologically relevant differences. Dunn’s post hoc test was applied to nonparametric data, and Tukey’s test to parametric data. In the comparative analyses between the SHR and Wistar lines, Two-Way ANOVA was applied. Analyses were performed using GraphPad Prism v8.0.0 for Windows (GraphPad Software, San Diego, CA, USA). Statistical significance was set at 5% (*p* ≤ 0.05). Data are expressed as mean ± standard deviation (SD) to represent the dispersion and biological variability within experimental groups.

## Results

### Flower and leaf extracts of *A. oleracea* show distinct cytotoxic and endocrine-modulating profiles in prostate cell lines

**Fig. 1:**
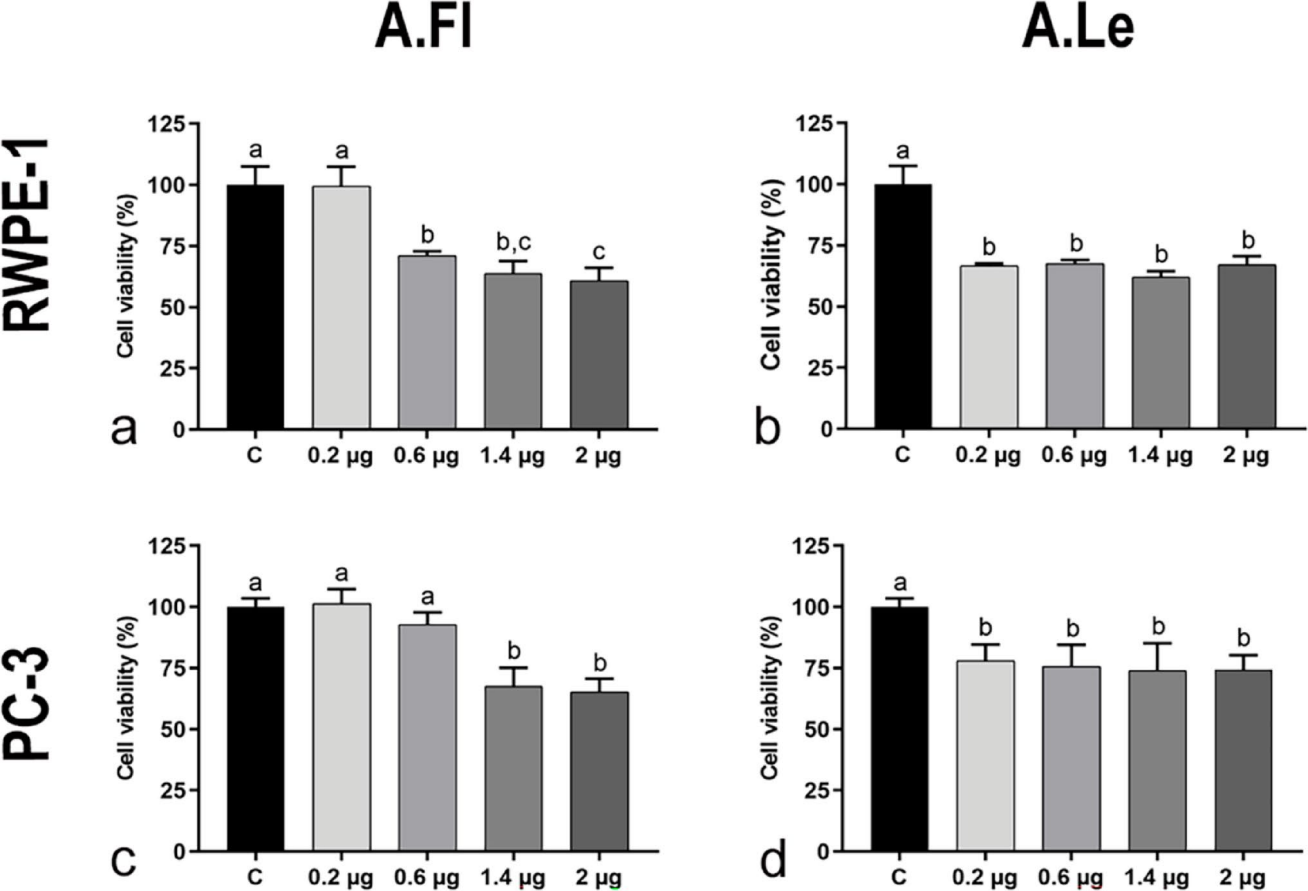
Cell viability of RWPE-1 (**a, b**) and PC-3 cell lines (**c, d**) after treatment with *A. oleracea* flower (A.Fl) and leaf (A.Le) extracts. The X-axis represents the μg/mL concentrations of the tested extracts. Values represent mean ± SD (*n* = 5/concentration, 24 h). Data were analyzed by ANOVA, except for PC-3/A.Le (Kruskal–Wallis). Different superscript letters indicate significant differences between treatments (*p* ≤ 0.05)

Treatment with *A. oleracea* extracts significantly reduced cell viability in both RWPE-1 (non-tumor) and PC-3 (tumor) prostate cell lines (Fig. 1). Exposure to the A.Le extract decreased cell viability at all tested concentrations (0.2, 0.6, 1.4, and 2 µg/mL) in both RWPE-1 and PC-3 cells (Fig. 1b, d). In contrast, A.Fl extract reduced RWPE-1 cell viability only at concentrations of 0.6, 1.4, and 2 µg/mL (Fig. 1a), while in PC-3 cells, the reduction was observed at 1.4 and 2 µg/mL (Fig. 1c). Given that A.Le extract reduced cell viability at the lowest concentration tested (0.2 µg/mL) in both cell lines, this concentration was selected as the standard for subsequent immunocytochemical analyses with both extracts.

**Fig. 2:**
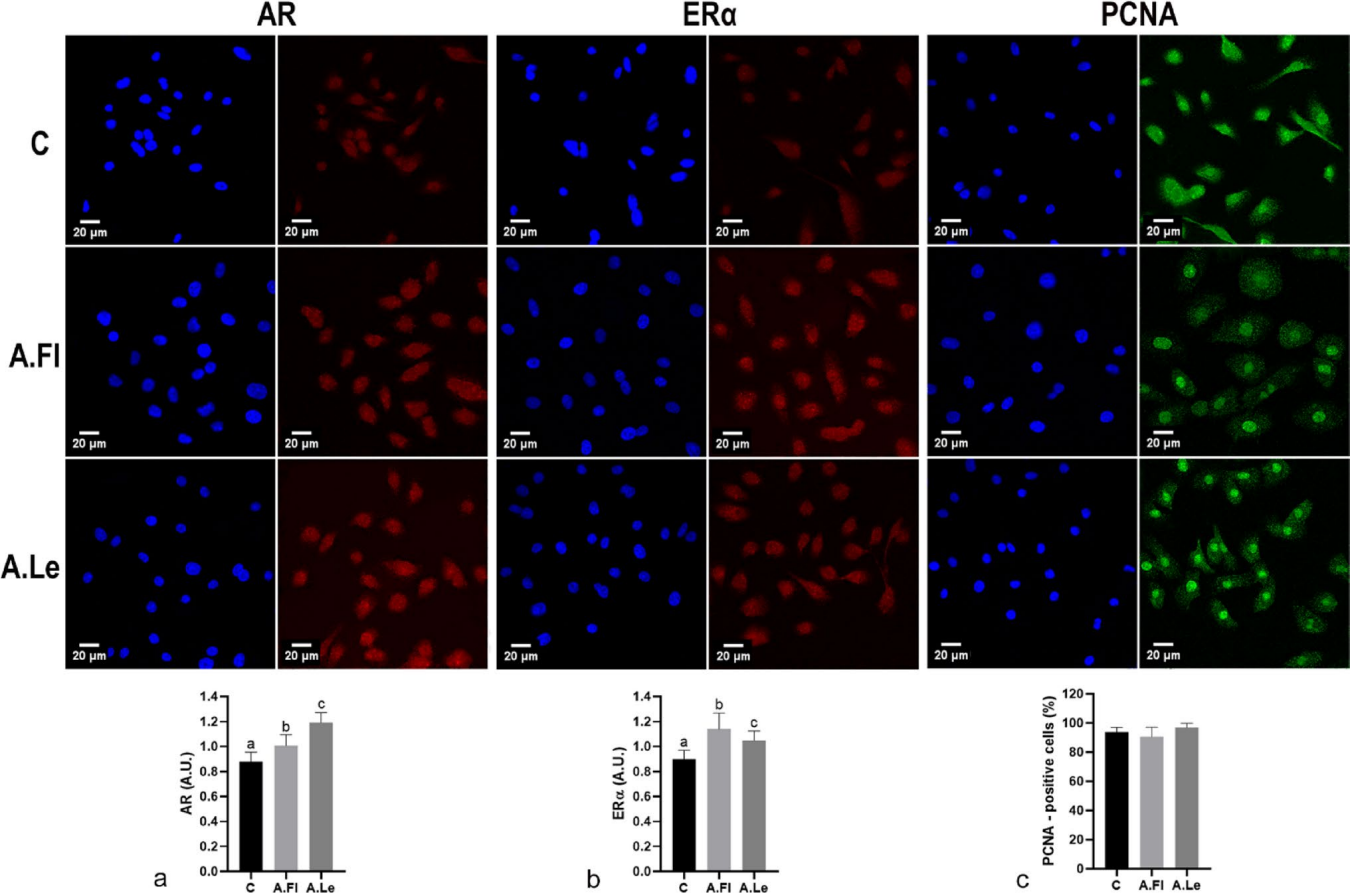
Immunofluorescence for AR (red), ERα (red), and PCNA (green) in RWPE-1 cells exposed to *A. oleracea* extracts. Nuclei are shown in blue color. Photomicrographs of immunocytochemical reactions from the control group (C) and cells treated with 0.2 µg/mL of flower (A.Fl) and leaf (A.Le) extracts (*n* = 5). Data are expressed as mean ± SD of Arbitrary Units (A.U.) for AR (**a**) and ERα (**b**), while PCNA (**c**) is presented as percentage (%). Superscript letters indicate significant differences among groups. Statistical analysis was performed using ANOVA/Tukey test, *p* ≤ 0.05

**Fig. 3:**
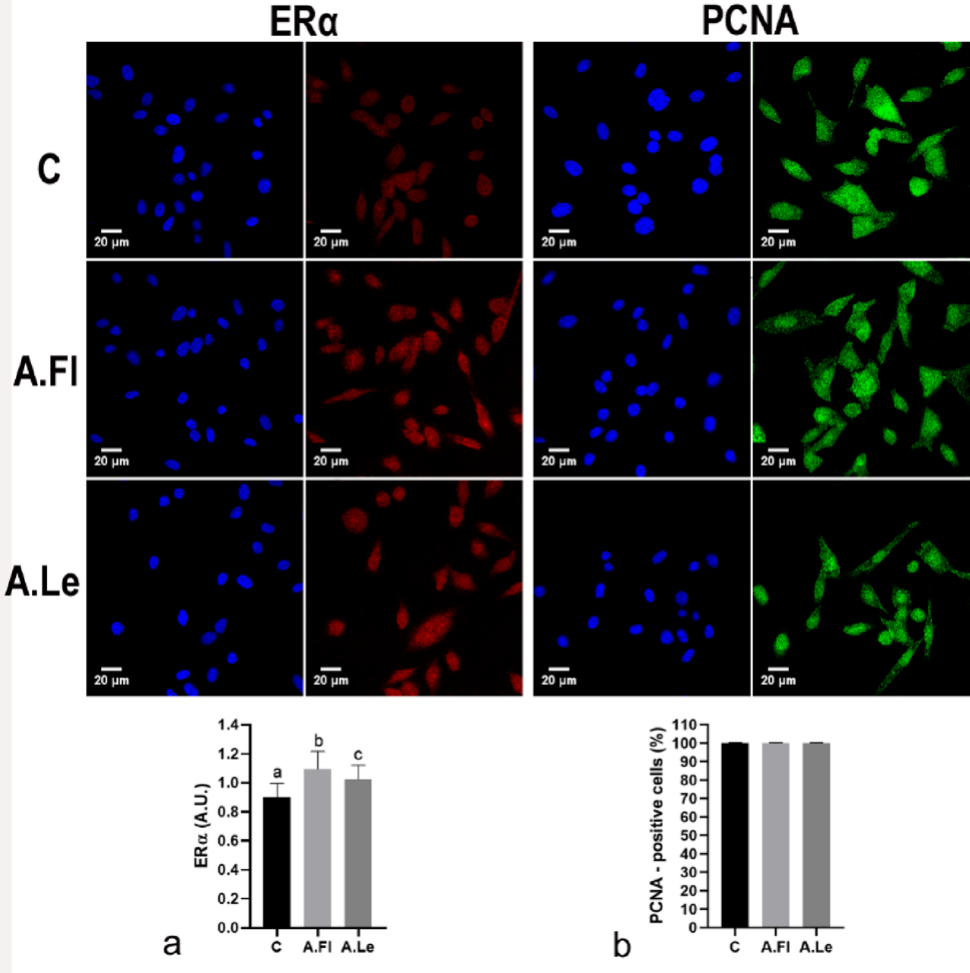
Immunofluorescence for ERα (red) and PCNA (green) in PC-3 cells exposed to *A. oleracea* extracts. Nuclei are shown in blue color. Photomicrographs of immunocytochemical reactions from the control group (C) and cells treated with 0.2 µg/mL of flower (A.Fl) and leaf (A.Le) extracts (*n* = 5). Data are expressed as mean ± SD of Arbitrary Units (A.U.) for ERα (**a**), and as percentage for PCNA (**b**). Superscript letters indicate significant differences among groups. Statistical analysis was performed using ANOVA/Tukey test, *p* ≤ 0.05

RWPE-1 cells exhibited increased immunolabeling for AR and ERα following exposure to both extracts (Fig. 2). However, A.Le induced a stronger AR upregulation (Fig. 2a), whereas A.Fl was more effective in upregulating ERα (Fig. 2b). No AR immunolabeling was detected in PC-3 cells, since this lineage does not express AR. However, an increase in ERα immunoreactivity was observed in both experimental groups, with this effect being more pronounced in the A.Fl group (Fig. 3a). No significant changes were detected in cell proliferation indices in either cell line under any treatment condition (Fig. 2c, 3b). These findings indicate that, at low concentrations, the extracts modulated steroid receptor expression without inducing proliferative or cytotoxic effects.

### At the systemic level, SHR treatment with A.Fl and A.Le resulted in persistent hypertension and differential modulation of hepatic and renal inflammation

A.Fl and A.Le did not affect the absolute or relative weights of the body, ventral prostate, liver, or kidneys in SHR (Table 1).

**Table 1:** Absolute (g) and relative weights of the body, ventral prostate, liver, and kidneys

	C	A.Fl	A.Le
*Weight (g)*			
Body	387.4 ± 36.6	371.0 ± 41.9	399.8 ± 17.0
Ventral prostate	0.5 ± 0.1	0.6 ± 0.1	0.6 ± 0.1
Liver	12.4 ± 1.3	12.5 ± 1.2	13.5 ± 1.0
Kidneys	2.5 ± 0.3	2.5 ± 0.3	2.6 ± 0.1
*Relative weight*			
Ventral prostate (× 10^–3^)	1.3 ± 0.1	1.5 ± 0.3	1.4 ± 0.2
Liver (× 10^–2^)	3.2 ± 0.1	3.4 ± 0.1	3.4 ± 0.2
Kidneys (× 10^–3^)	6.5 ± 0.5	6.7 ± 0.5	6.6 ± 0.3

Data are expressed as mean ± standard deviation (*n* = 8 animals/group). All data showed parametric distribution and no significant variation among groups

**Fig. 4:**
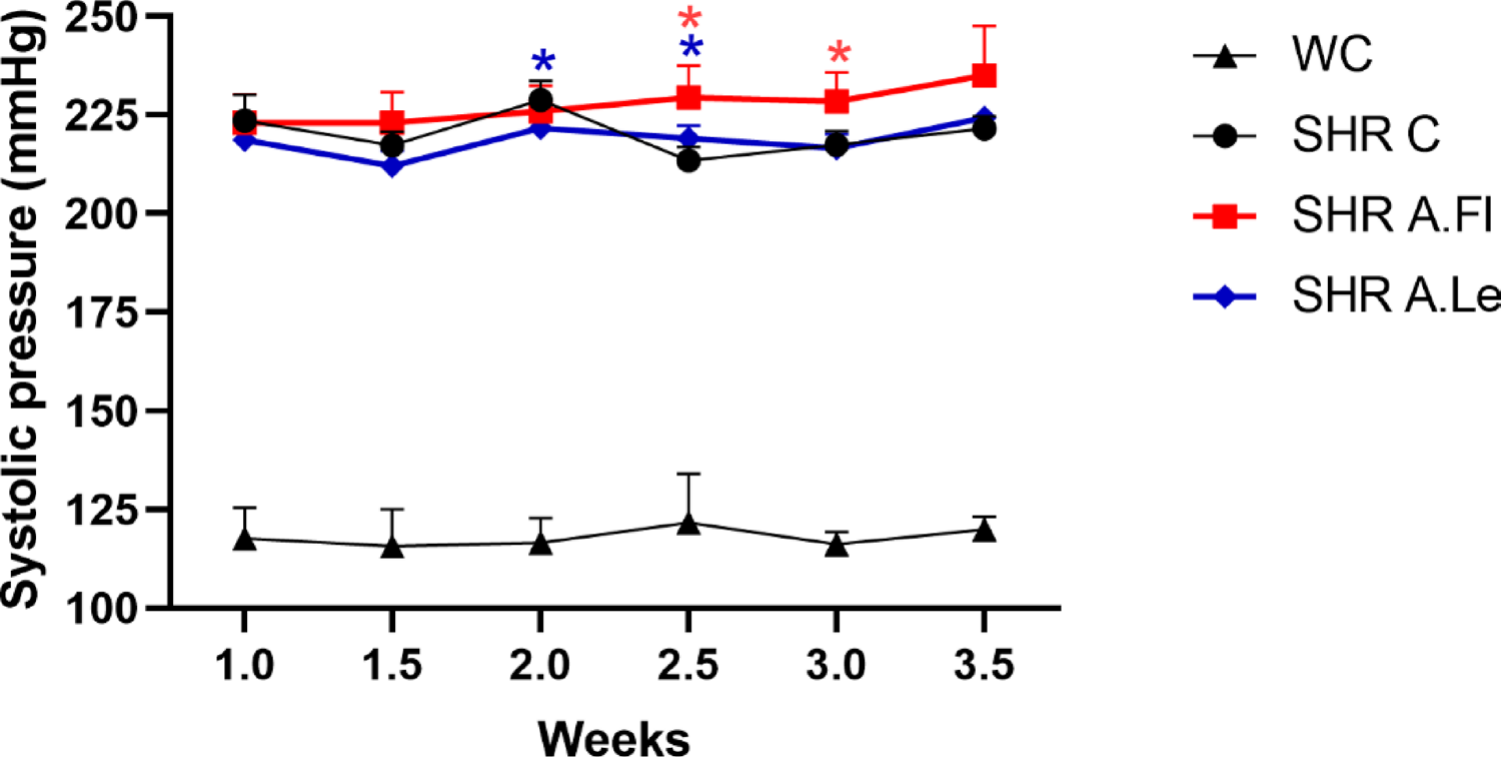
Variation of systolic blood pressure in SHR and Wistar strains. The Wistar strain was used as the normotensive control for blood pressure. Data are presented as mean ± SD (*n* = 8 animals /group). Statistical analysis was performed using two-way ANOVA, *p* ≤ 0.05)

Both *A. oleracea* extracts failed to reduce blood pressure in SHR (Fig. 4). Although A.Fl and A.Le induced fluctuations in blood pressure throughout the treatment, after 21 days of exposure, SHR maintained elevated blood pressure levels comparable to the control group.

**Fig. 5:**
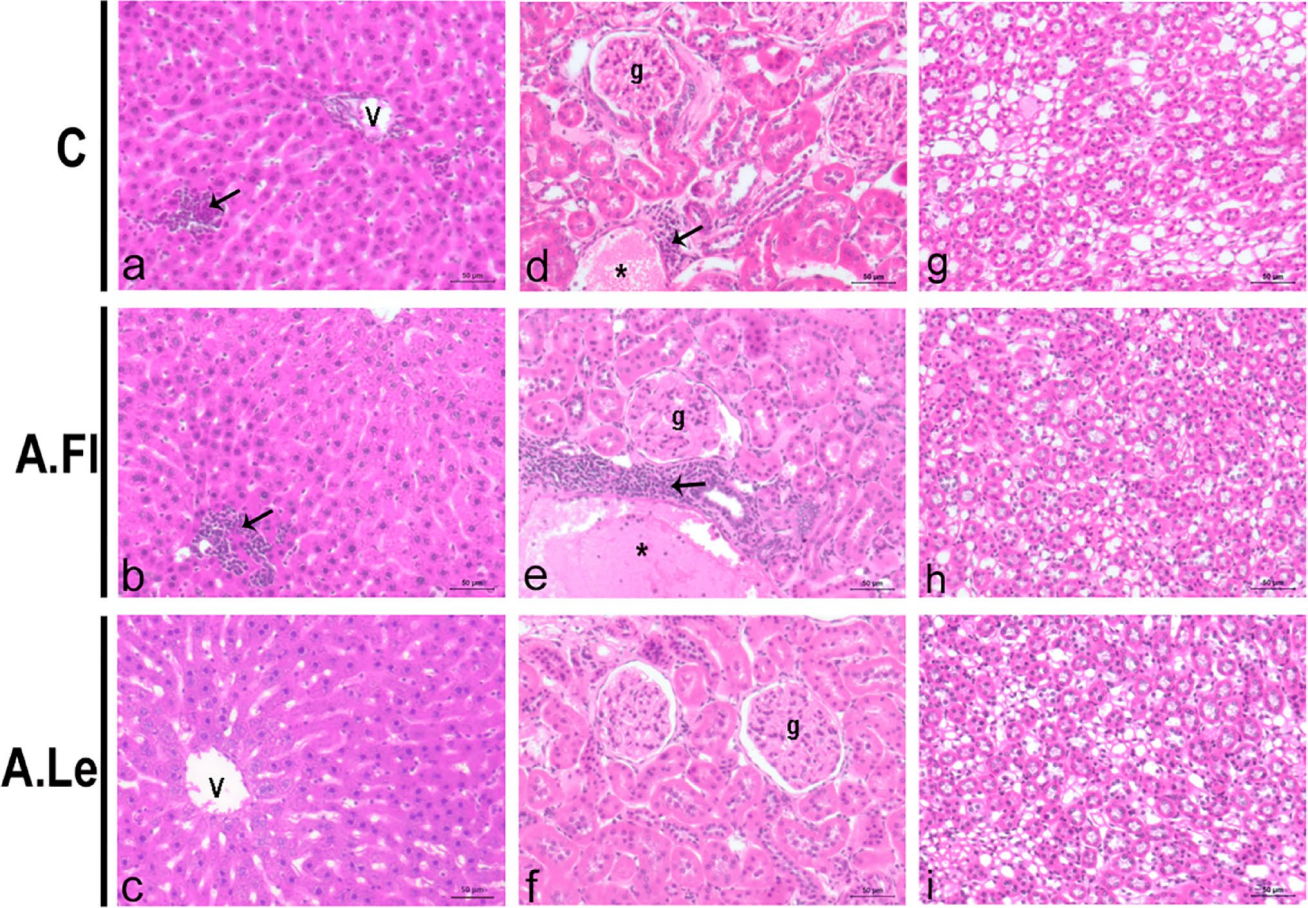
Histological sections of the liver (**a**–**c**), renal cortex (**d**–**f**), and renal medulla (**g**–**i**) stained with HE (*n* = 5 animals/group). Arrows indicate inflammatory infiltrates and asterisks (*) highlight edematous lymphatic vessels in the renal cortex. Central vein (v); renal glomerulus (g)

SHRC exhibited inflammatory foci in the hepatic parenchyma (Fig. 5a). In addition, the renal cortex of these animals showed diffuse interstitial inflammation. However, no histopathological alterations were observed in the renal medulla (Fig. 5d, g). Treatment with A.Fl did not reverse the inflammatory profile observed in the liver and kidneys of SHRC (Fig. 5b, e, h). In contrast, A.Le attenuated inflammatory foci in both the liver and renal cortex (Fig. 5c, f, i).

### Prostatic hyperplasia in SHR was alleviated by both extracts, with A.Fl showing greater effectiveness

**Fig. 6:**
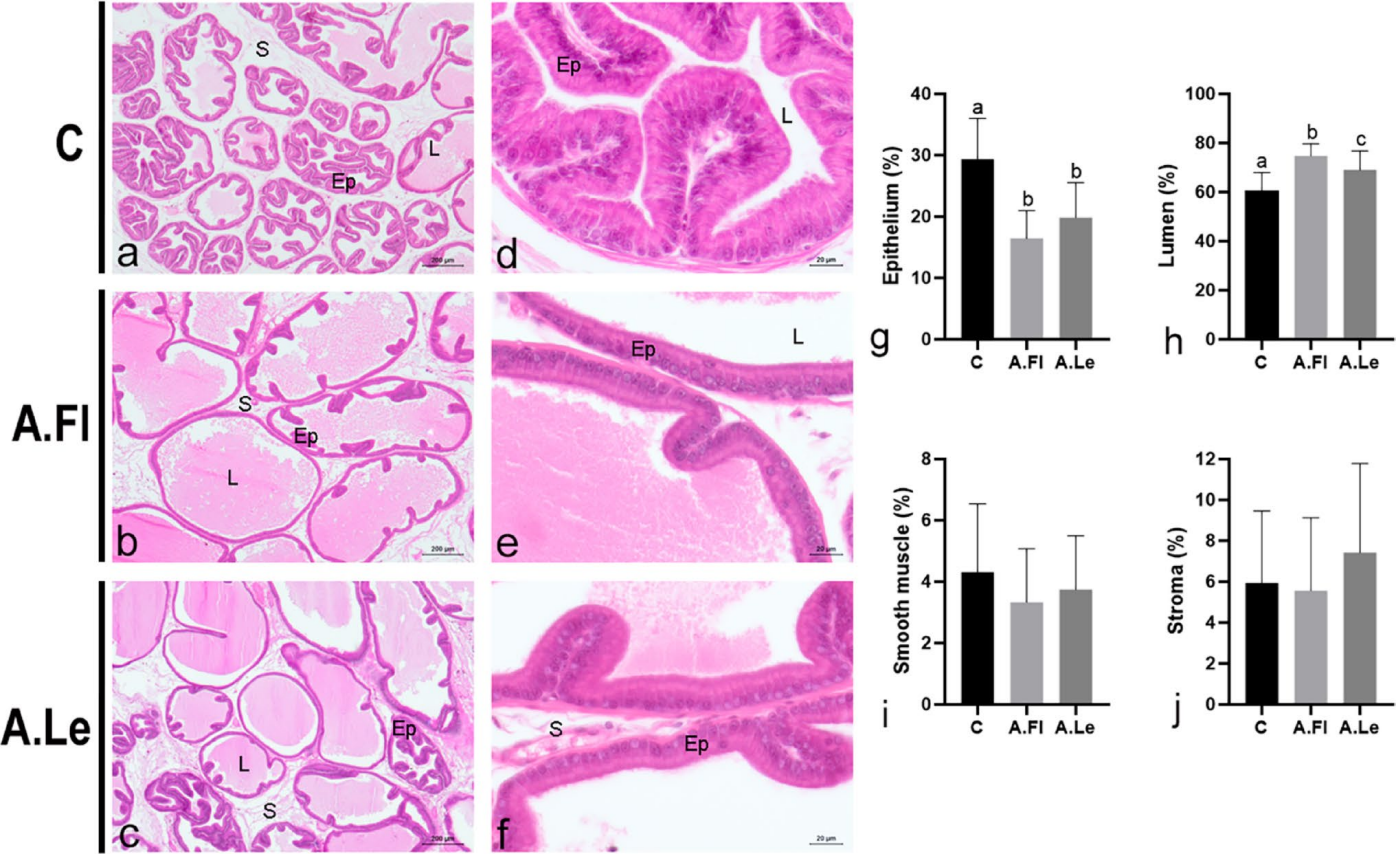
Histology and stereology of the ventral prostate HE (*n* = 5 animals/group). **a–f** HE–stained histological sections. Epithelium (Ep), lumen (L), stroma (S). **g–j** Relative frequency (%) of tissue compartments in the ventral prostate. Data are expressed as mean ± SD (*n* = 30 fields/group). Statistical analysis was performed by ANOVA/Tukey (**g**, **h**) and Kruskal–Wallis (**i**, **j**). Superscript letters indicate statistical differences among groups, *p* ≤ 0.05

The ventral prostate of SHRC exhibited marked epithelial hyperplasia, characterized by papillary projections extending into the lumen (Fig. 6a, d). A.Fl and A.Le reduced the frequency of these papillary epithelial projections, resulting in a more linear epithelial profile (Fig. 6b–c, e–f). Histopathological analysis indicated that both extracts attenuated the hyperplastic phenotype of the SHR prostate (Table 2). A.Fl was more effective in decreasing the frequency of alveoli with severe hyperplasia, thereby increasing the proportion of normal alveoli and those with mild/moderate hyperplasia. Conversely, A.Le significantly increased the frequency of normal alveoli but did not substantially reduce the proportion of alveoli with mild/moderate or severe hyperplasia. Stereological analysis demonstrated that both A.Fl and A.Le reduced the relative frequency (%) of the epithelium and increased the luminal area, with A.Fl showing greater efficacy in expanding the prostatic lumen (Fig. 6g–j). Smooth muscle and stromal compartments were not affected by the treatments.

**Table 2:** Frequency (%) of normal alveoli, alveoli with mild/moderate hyperplasia, and alveoli with severe hyperplasia in the ventral prostate of SHR

	C	A.Fl	A.Le
*Alveoli profile (%)*			
Normal	4.6 ± 2.5^a^	12.9 ± 5.8^b^	8.0 ± 4.1^c^
Mild/Moderate hyperplasia	45.9 ± 12.7^a^	68.52 ± 12.3^b^	53.8 ± 12.9^a^
Intense hyperplasia	49.5 ± 12.7^a^	18.6 ± 14.6^b^	38.3 ± 14.9^a^

Data are presented as mean ± standard deviation. Superscript letters indicate significant differences among groups, *p* ≤ 0.05. ANOVA/Tukey test was used for normal alveoli, and Kruskal–Wallis/Dunn for mild/moderate and severe hyperplasia

**Fig. 7:**
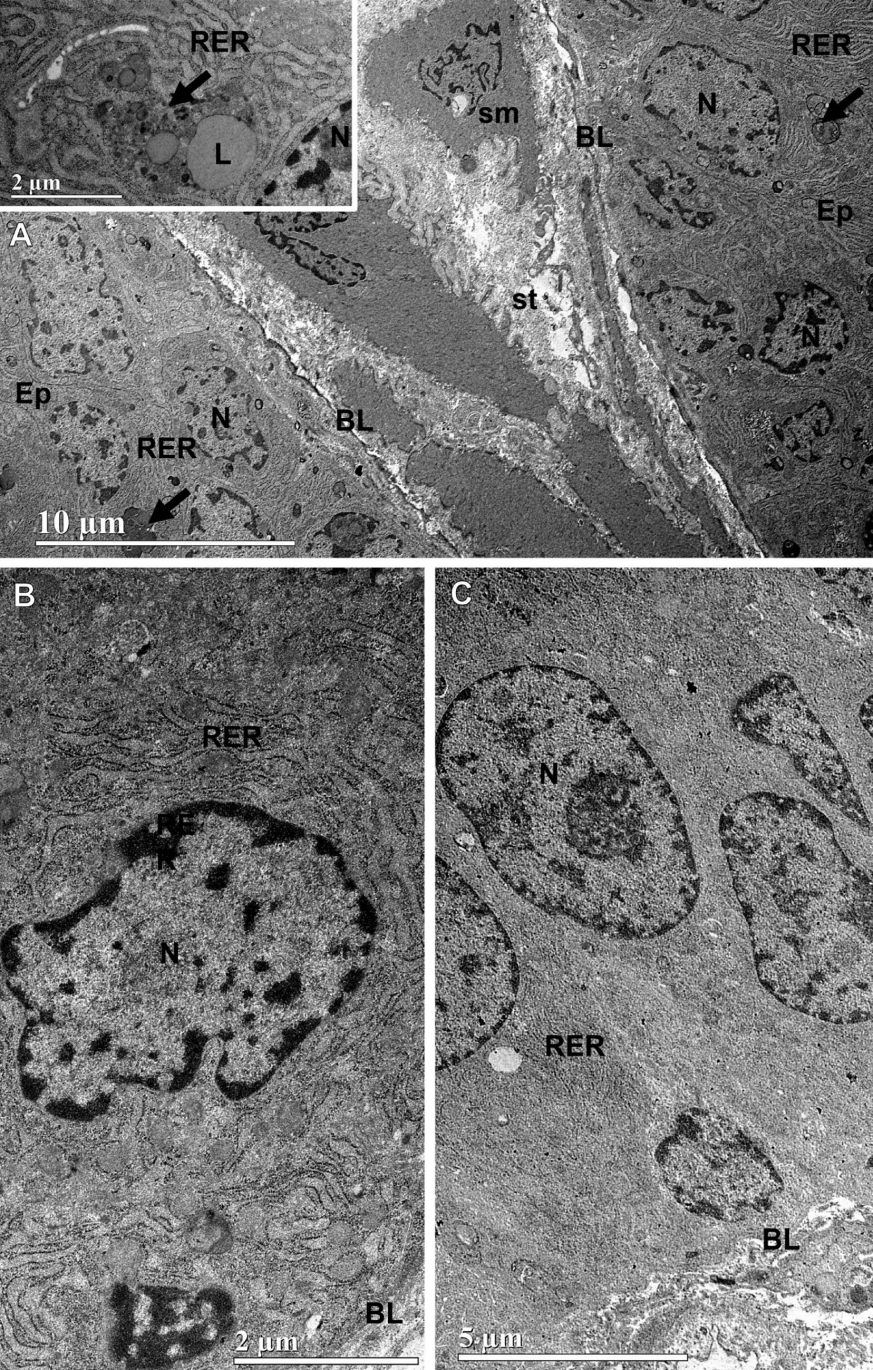
Ultrastructure of the ventral prostate (*n* = 3 animals/group). In SHRC (panel A), the stratified epithelium (Ep) showed low cuboidal cells with pleomorphic nuclei (N). The cytoplasm contained abundant rough endoplasmic reticulum (RER) and numerous supranuclear multivesicular bodies (arrows). The stroma (st) exhibited smooth muscle cells (sm) embedded in fibrous connective tissue. Basal lamina (BL). The inset in panel **A** shows a supranuclear cytoplasmic region with a multivesicular body associated with lipid inclusions (L). Panel **B** depicts a columnar epithelial cell from A.Fl, with cytoplasm rich in RER and an irregularly shaped nucleus (N). Panel **C** shows columnar epithelium from A.Le, with elongated and regular nuclei, and absence of multivesicular bodies

At the ultrastructural level, the hyperplastic pattern of the ventral prostate in SHRC was characterized by epithelial stratification, evidenced by the stacking of cells with irregular nuclei (Fig. 7A). Epithelial cells exhibited cytoplasm rich in rough endoplasmic reticulum and numerous multivesicular bodies associated with lipid inclusions. Nuclear pleomorphism was prominent, with irregular shapes and sizes. The stroma consisted predominantly of smooth muscle cells and connective fibers. Treatments with A.Fl (Fig. 7B) and A.Le (Fig. 7C) attenuated epithelial stratification. Neither extract altered the biosynthetic-secretory organelles; however, both reduced the frequency of multivesicular bodies. Moreover, A.Le markedly decreased nuclear pleomorphism, yielding more regular epithelial nuclei.

**Fig. 8:**
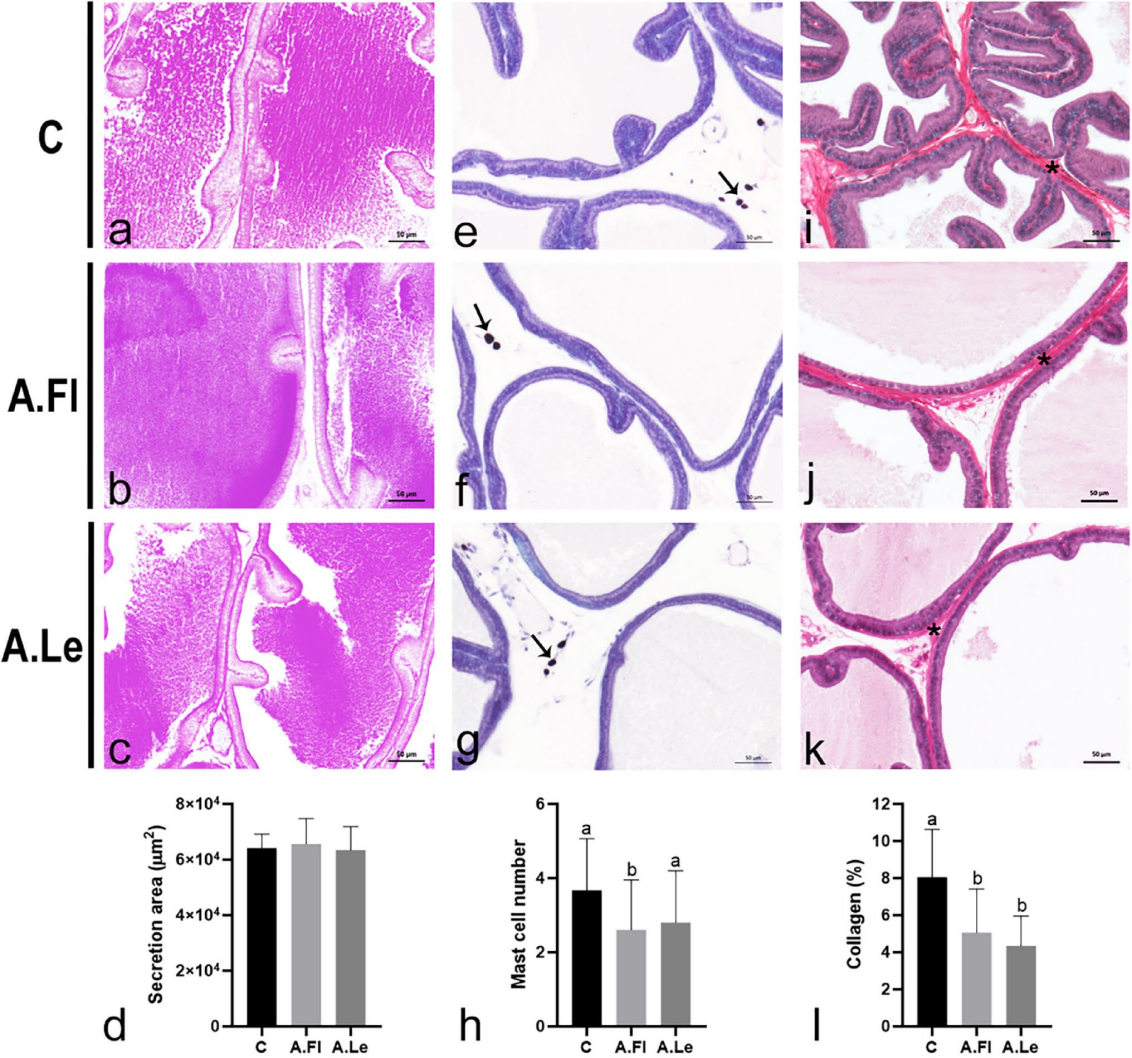
PAS (**a-c**), toluidine blue (**e-g**), and picrosirius–hematoxylin staining (**i-k**) of the ventral prostate. Arrows and asterisks indicate mast cells and collagenous material, respectively. Graphs represent glandular secretion area (**d**), mast cell count (**h**), and collagen fiber frequency (**l**). Data are expressed as mean ± SD (30 fields; *n* = 5 animals/group). Statistical analysis was performed by ANOVA (**l**) and Kruskal–Wallis (**d**, **h**). Superscript letters indicate significant differences among groups, *p* ≤ 0.05

Neither A.Fl nor A.Le altered the amount of PAS-positive secretion within the glandular lumen (Fig. 8a–d). Nevertheless, a significant reduction in mast cell numbers was observed in the A.Fl group (Fig. 8e–h). In addition, both A.Fl and A.Le similarly reduced collagen fiber content in the prostatic stroma (Fig. 8i–l).

### Modulation of AR activity and proliferation by *A. oleracea* extracts in SHR prostate occurs independently of microvascular or antioxidant pathways

**Fig. 9:**
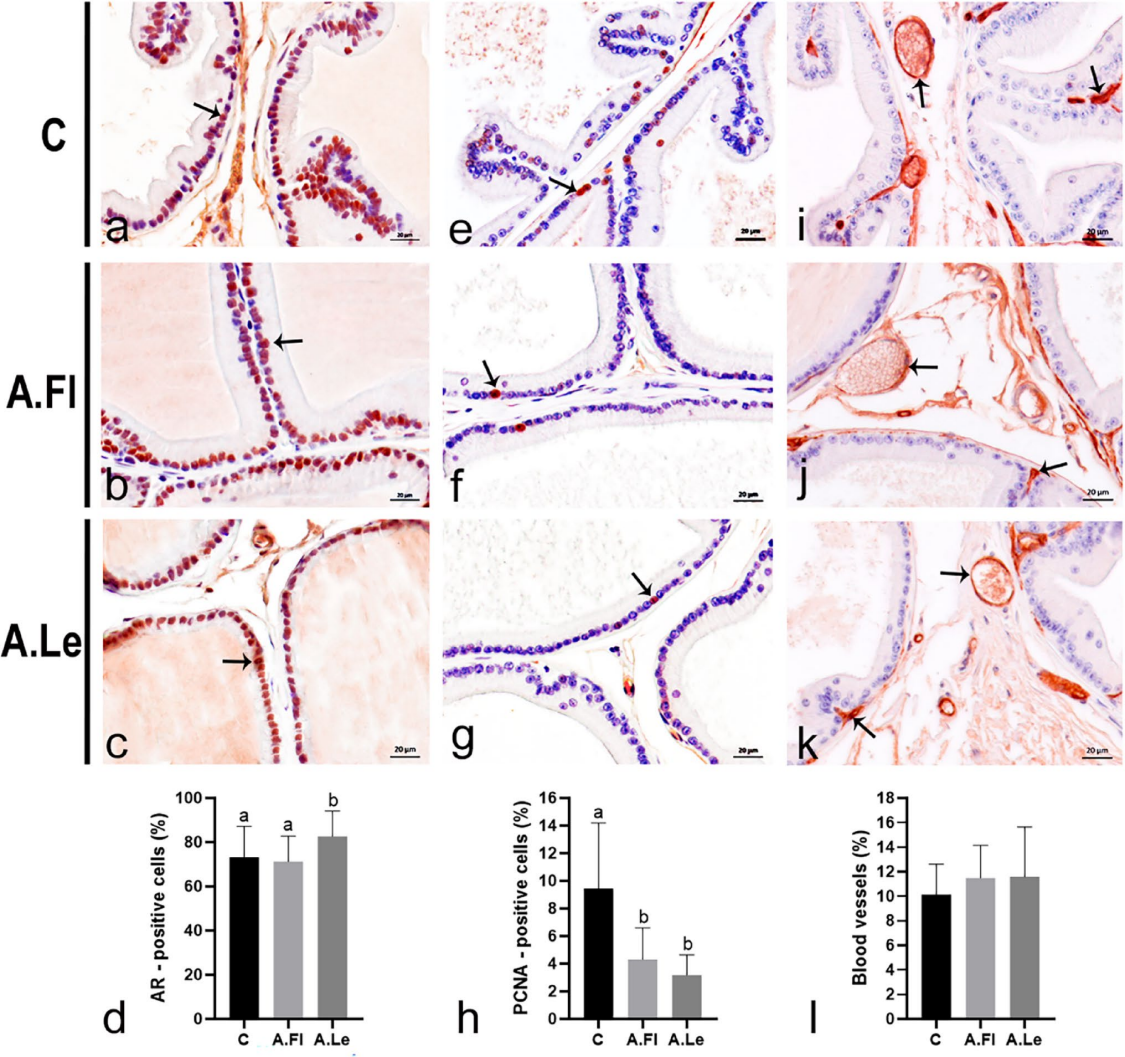
Immunohistochemical staining (arrows) for AR (**a**–**c**), PCNA (**e**–**g**), and Aquaporin-1 (**i**–**k**); hematoxylin counterstaining. Graphs show quantification of AR-positive (**d**) and PCNA-positive (**h**) cells, as well as the frequency (%) of blood vessels per microscopical field (**l**). Data are expressed as mean ± SD (30 fields; *n* = 5 animals/group). Statistical analysis was performed by Kruskal–Wallis test. Superscript letters indicate significant differences among groups, *p* ≤ 0.05

A.Le increased AR immunostaining in epithelial cells compared with SHRC and A.Fl groups (Fig. 9a–d). In addition, both extracts significantly reduced cell proliferation (Fig. 9e–h). The potential of A.Fl and A.Le to improve prostatic vascularization was assessed by AQP1 immunostaining (Fig. 9i–k). No significant differences were observed in AQP1-positive area (C = 1,159.64 ± 337.00 µm^2^; A.Fl = 1,160.85 ± 289.20 µm^2^; A.Le = 998.65 ± 212.90 µm^2^), nor in blood vessel frequency (Fig. 9l). Finally, the investigation of oxidative stress pathways revealed that neither A.Fl nor A.Le exerted antioxidant effects in the ventral prostate of SHR. CAT activity was reduced in the A.Fl group, with no changes in SOD, MDA, or CP levels. In contrast, A.Le increased MDA levels without altering CAT, SOD, or CP (Table 3).

**Table 3:** Quantitative data for catalase (CAT), superoxide dismutase (SOD), malondialdehyde (MDA), and carbonyl protein (CP)

	C	A.Fl	A.Le
CAT	4.9 ± 1.6^a^	3.0 ± 1.1^b^	3.7 ± 1.2^a^
SOD	38.2 ± 8.5	37.4 ± 13.9	43.8 ± 12.1
MDA	2.7 ± 0.3^a^	3.0 ± 0.6^a^	4.3 ± 1.5^b^
CP	4.4 ± 0.8	5.1 ± 1.7	4.7 ± 0.6

Data are expressed as mean ± standard deviation (*n* = 8 animals/group). Statistical analysis was performed by ANOVA (CAT) and Kruskal–Wallis (SOD, MDA, CP). Superscript letters indicate significant differences among groups, *p* ≤ 0.05

## Discussion

This study employed SHR and the human prostate cell lines RWPE-1 and PC-3 as experimental models to investigate the biological activities of *A. oleracea* flower (A.Fl) and leaf (A.Le) extracts. The biological effects of *A. oleracea* observed in this study can be interpreted in light of the known mechanisms of its major bioactive constituent, spilanthol, together with associated minor compounds. Spilanthol has been shown to modulate steroid hormone signaling, inflammatory pathways, and cellular redox balance, all of which play central roles in the pathogenesis of BPH. In the prostate, these mechanisms may converge to regulate epithelial proliferation, stromal remodeling, and tissue homeostasis, thereby providing a mechanistic framework for the anti-hyperplastic effects observed in vivo.

RWPE-1 and PC-3 cells were employed as in vitro models to assess prostate cell viability in response to exposure to *A. oleracea* extracts. Under the experimental conditions used, A.Fl exhibited lower cytotoxicity at low concentrations in both RWPE-1 (0.2 μg) and PC-3 cells (0.2–0.6 μg), whereas all concentrations of A.Le tested induced cytotoxic effects in both cell lines.

These results indicate that both extracts are capable of reducing cell viability in PC-3 prostate cancer cells; however, this effect was not selective, as comparable cytotoxic responses were also observed in non-tumor prostate epithelial RWPE-1 cells. Although RWPE-1 cells are widely used as a non-tumorigenic prostate epithelial model, they do not fully recapitulate the cellular heterogeneity, stromal interactions, and hormonal microenvironment of the normal human prostate, which limits direct translational extrapolation (Dent et al. 2019; Moya et al. 2023). This limitation may partly account for the variability in cytotoxic responses observed across experimental systems.

Similar context-dependent cytotoxic effects of *A. oleracea* extracts have been reported in other cellular models. For example, anticancer activity has been described in gastric carcinoma cells (AGP01) treated with hydroethanolic flower extracts and spilanthol at concentrations ranging from 1.25 to 80 μg/mL (Pinheiro et al. 2023). In contrast, methanolic extracts from flowers, leaves, and stems induced dose-dependent cell death in normal human embryonic kidney cells (HEK-293) at higher concentrations (10–400 μg/mL) (Gerbino et al. 2016), whereas no significant cytotoxicity was observed in rat vascular smooth muscle cells (A7r5) treated with ethanolic extracts (25–100 μg/mL) (Stein et al. 2021).

Collectively, these findings support the notion that the cytotoxic effects of *A. oleracea* extracts are highly dependent on concentration, extraction method, and cell type. In the prostate cell lines analyzed here, A.Fl consistently displayed a more favorable cytotoxicity profile compared with A.Le. Importantly, the cytotoxic effects observed in vitro should not be interpreted as evidence of selective anticancer activity, but rather as concentration-dependent cellular responses obtained under simplified experimental conditions. The concentrations associated with cytotoxicity were higher than those used to evaluate endocrine receptor modulation and anti-hyperplastic effects. To further strengthen translational relevance, additional selectivity studies using stromal fibroblasts or primary prostate cells will be required to better define tissue-specific responses and therapeutic margins.

Moreover, the in vitro concentrations employed were selected to probe cellular sensitivity and do not directly reflect systemic exposure levels achieved in vivo, where absorption, tissue distribution, metabolism, and clearance substantially influence bioavailability. Consistent with this interpretation, in vivo treatment with *A. oleracea* extracts did not induce histopathological alterations in the prostate, liver, or kidneys, indicating a favorable safety profile under the experimental conditions employed.

Accordingly, the therapeutic relevance of *A. oleracea* extracts appears to lie primarily in their regulatory and tissue-modulating properties rather than in selective cytotoxicity. Future dose–response and pharmacokinetic studies will be required to further define the therapeutic window and optimize translational applicability.

Beyond their effects on cell viability, and consistent with their proposed regulatory role, the extracts also exerted marked actions on steroid hormone receptors. To our knowledge, this is the first demonstration of the regulatory effects of *A. oleracea* on hormonal receptors in both in vitro and in vivo models. Our findings revealed that in RWPE-1 cells exposed to a low concentration of *A. oleracea* (0.2 μg/mL) AR modulation was more strongly induced by A.Le, whereas ERα was preferentially upregulated by A.Fl. In PC-3 cells, which lack AR expression, exposure to A.Fl also resulted in marked increase in ERα immunostaining. These results indicate that A.Fl and A.Le act through distinct hormonal pathways, with A.Fl eliciting more pronounced effects on ERα, whereas A.Le exerts stronger action on AR. Despite these divergent patterns of AR and ERα upregulation, neither extract altered cell proliferation in vitro.

The potential of A.Le to modulate AR signaling was supported in vivo, as only this extract increased AR immunostaining in the ventral prostate of SHR, corroborating its androgenic profile previously observed in vitro. However, increased AR immunostaining reflects receptor abundance rather than functional transcriptional activity, which was not directly assessed in this study. Accordingly, the absence of downstream AR target analyses, such as PSA, NKX3.1, or cell cycle regulators, represents a limitation. Despite this, the present findings provide the first direct evidence that *A. oleracea* can modulate AR expression, extending earlier reports that only indirectly suggested endocrine activity through increased testosterone levels and improved sexual function (Sharma et al. 2011; Patnaik et al. 2022).

Notably, attenuation of prostatic hyperplasia was not strictly associated with AR activation. The A.Fl, which did not increase AR expression in vivo, produced the most pronounced reduction in epithelial hyperplasia, indicating that the anti-hyperplastic effects of *A. oleracea* are mediated predominantly through AR-independent mechanisms. These effects likely involve ERα signaling, anti-inflammatory actions, and stromal remodeling. In this context, ERα modulation by A.Fl may counterbalance androgen-driven proliferative signaling through AR-ERα cross-talk, thereby promoting epithelial growth control and stromal homeostasis. Although ERα signaling can exert both proliferative and anti-proliferative effects depending on cellular context, ligand balance, and disease stage (Lafront et al. 2024), the present data suggest a growth-regulatory role under the experimental conditions employed.

Consistent with this interpretation, both extracts attenuated prostatic hyperplasia in SHR; however, A.Fl, which did not alter AR expression, exhibited a more favorable anti-hyperplastic profile. In the ventral prostate, A.Fl reduced epithelial proliferation while preserving secretory features and was associated with decreased collagen deposition and mast cell infiltration. At the ultrastructural level, epithelial cells displayed a typical secretory phenotype without nuclear pleomorphism or signs of cellular senescence. Given that AR activation is a major driver of prostatic growth and BPH progression (Vickman et al. 2020; Ren et al. 2024), phytochemicals that do not directly enhance AR signaling, as observed for A.Fl, may represent more promising candidates for modulating prostatic hyperplasia.

The SHR is a well-established experimental model that develops prostatic hyperplasia as a secondary consequence of chronic hypertension, recapitulating several pathophysiological features associated with BPH in humans, including endocrine imbalance, tissue hypoxia, chronic inflammation, and oxidative stress (Saito et al. 2014; Shimizu et al. 2015; Kyoda et al. 2019). In the present study, the attenuation of prostatic hyperplasia occurred in the absence of any reduction in systemic blood pressure, which strengthens the interpretation that the effects of *A. oleracea* are mediated by direct, tissue-specific actions within the prostate rather than by indirect antihypertensive mechanisms.

Although anatomical differences exist between species, the rat ventral prostate shares key histological, hormonal, and stromal regulatory characteristics with the human periurethral prostate, the primary site affected in BPH (Zhang et al. 2021). Accordingly, morphological and molecular changes observed in the ventral prostate of SHR are considered relevant for translational interpretation of epithelial-stromal interactions and hyperplastic processes in human disease. Taken together, these findings indicate that the lack of antihypertensive effects should not be viewed as a negative outcome but rather as evidence of prostate-specific pharmacological activity, reinforcing the translational value of the SHR model for evaluating tissue-targeted therapeutic strategies for BPH.

Chronic inflammation and stromal remodeling are key drivers of BPH, and mast cells play a central role in this process by promoting fibrosis, extracellular matrix deposition, and epithelial-stromal cross-talk (Barron and Rowley 2012; De Nunzio et al. 2020; Pattabiraman et al. 2021). In the present study, A.Fl markedly reduced mast cell infiltration and collagen deposition in the ventral prostate of SHR, indicating attenuation of a pro-fibrotic and pro-inflammatory stromal microenvironment. Given the established association between mast cells, stromal fibrosis, and hyperplastic progression in the prostate, these findings support a mechanistic link between anti-inflammatory stromal modulation and the observed reduction in epithelial hyperplasia (Ou et al. 2017).

Notably, the anti-inflammatory effects of *A. oleracea* were tissue-specific. While A.Le reduced inflammatory foci in the liver and kidneys, only A.Fl decreased mast cell density in the prostate. This tissue specificity likely reflects differences in local immune context, stromal composition, receptor expression, and extract bioavailability across organs. Importantly, modulation of the prostatic stromal inflammatory niche by A.Fl provides a plausible mechanism by which epithelial proliferation is indirectly restrained, reinforcing the concept that the anti-hyperplastic effects of *A. oleracea* are mediated, at least in part, through regulation of epithelial-stromal interactions rather than direct effects on epithelial cells alone.

To assess whether redox modulation contributed to the anti-hyperplastic effects observed in this study, oxidative stress parameters were evaluated in the ventral prostate of SHR. A.Fl reduced CAT activity without altering CP levels or lipid peroxidation, whereas A.Le increased lipid peroxidation without significantly affecting antioxidant enzyme activities. These findings indicate that antioxidant mechanisms are unlikely to represent the primary pathway underlying the attenuation of prostatic hyperplasia induced by *A. oleracea* extracts.

Although *A. oleracea* has been widely reported to exhibit antioxidant properties in different experimental settings, redox responses to phytochemicals are highly context-dependent and influenced by plant organ, extraction method, dose, and target tissue (Wongsawatkul et al. 2008; Prachayasittikul et al. 2013; Nascimento et al. 2020). The pro-oxidant profile observed with A.Le under the present experimental conditions may reflect a biphasic or hormetic response, which has been described for several bioactive plant compounds and does not necessarily imply overt tissue damage.

Accordingly, the oxidative stress data are best interpreted as a limitation with respect to defining redox-dependent mechanisms of action. Importantly, the attenuation of prostatic hyperplasia observed here occurred largely independently of antioxidant effects, distinguishing *A. oleracea* from phytotherapeutic agents whose efficacy relies primarily on redox modulation.

The *A. oleracea* extracts employed in this study were composed predominantly of the N-alkylamide spilanthol, as previously reported in phytochemical analyses (Peretti et al. 2021; Rodrigues et al. 2023). Although spilanthol was the major constituent in both extracts, the A.Fl composition included, in addition to 97.7% spilanthol, minor fractions of scopoletin (1.53%) and d-limonene (0.77%)—compounds with well-documented biological activities, including antioxidant, anti-inflammatory, and antimicrobial properties (Sun 2007; Gao et al. 2024). The combined presence of these metabolites may exert additive or synergistic effects with spilanthol, which could explain the greater efficacy of A.Fl compared with A.Le, in which spilanthol was virtually the sole compound detected (98.5%). Thus, although spilanthol accounts for most of the pharmacological properties attributed to *A. oleracea* (Barbosa et al. 2016), the contribution of minor constituents in A.Fl likely enhances and broadens its biological activity, thereby providing a plausible explanation for the superior outcomes observed in this study.

## Conclusion

In conclusion, both *A. oleracea* flower and leaf extracts attenuated prostatic hyperplasia in SHR, although the flower extract exhibited a more favorable biological profile. Importantly, the present findings should be interpreted as evidence of distinct biological and endocrine-modulatory effects rather than as an indication of immediate therapeutic applicability. The differential modulation of androgen and estrogen receptors, the non-selective cytotoxicity observed in vitro, the pro-oxidant profile associated with the leaf extract, and the lack of downstream functional evaluation of AR signaling collectively underscore the need for cautious interpretation. Together, these results provide proof of concept that different plant organs of *A. oleracea* exert divergent effects on prostate biology, supporting further mechanistic and translational investigations.

## Data Availability

The data that support the findings of this study are available from the corresponding author upon reasonable request.
